# Advances in gene ontology utilization improve statistical power of annotation enrichment

**DOI:** 10.1371/journal.pone.0220728

**Published:** 2019-08-15

**Authors:** Eugene W. Hinderer, Robert M. Flight, Rashmi Dubey, James N. MacLeod, Hunter N. B. Moseley

**Affiliations:** 1 Department of Molecular and Cellular Biochemistry, University of Kentucky, Lexington, KY, United States of America; 2 Markey Cancer Center, University of Kentucky, Lexington, KY, United States of America; 3 Maxwell H. Gluck Equine Research Center, University of Kentucky, Lexington, KY, United States of America; 4 Department of Veterinary Science, University of Kentucky, Lexington, KY, United States of America; 5 Institute for Biomedical Informatics, University of Kentucky, Lexington, KY, United States of America; The University of Tokyo, JAPAN

## Abstract

Gene-annotation enrichment is a common method for utilizing ontology-based annotations in gene and gene-product centric knowledgebases. Effective utilization of these annotations requires inferring semantic linkages by tracing paths through edges in the ontological graph, referred to as relations. However, some relations are semantically problematic with respect to scope, necessitating their omission or modification lest erroneous term mappings occur. To address these issues, we created the Gene Ontology Categorization Suite, or GOcats—a novel tool that organizes the Gene Ontology into subgraphs representing user-defined concepts, while ensuring that all appropriate relations are congruent with respect to scoping semantics. Here, we demonstrate the improvements in annotation enrichment by re-interpreting edges that would otherwise be omitted by traditional ancestor path-tracing methods. Specifically, we show that GOcats’ unique handling of relations improves enrichment over conventional methods in the analysis of two different gene-expression datasets: a breast cancer microarray dataset and several horse cartilage development RNAseq datasets. With the breast cancer microarray dataset, we observed significant improvement (one-sided binomial test p-value = 1.86E-25) in 182 of 217 significantly enriched GO terms identified from the conventional path traversal method when GOcats’ path traversal was used. We also found new significantly enriched terms using GOcats, whose biological relevancy has been experimentally demonstrated elsewhere. Likewise, on the horse RNAseq datasets, we observed a significant improvement in GO term enrichment when using GOcat’s path traversal: one-sided binomial test p-values range from 1.32E-03 to 2.58E-44.

## Introduction

### Ontologies and gene set enrichment analyses

Biological and biomedical ontologies such as Gene Ontology (GO) [1] are indispensable tools for systematically annotating genes and gene products using a consistent set of annotation terms. Ontologies are used to document new knowledge gleaned from nearly every facet of biological and biomedical research today, from classic biochemical experiments elucidating specific molecular players in disease processes to omics-level experiments providing systemic information on tissue-specific gene regulation. These ontologies are created, maintained, and extended by experts with the goal of providing a unified annotation scheme that is readable by humans and machines [2]. With the advent of transcriptomics technologies, high-throughput investigation of the functional impact of gene expression in biological and disease processes in the form of gene set enrichment analyses represents one important use of GO [3]. Many different tools such as Categorizer [4], GOATOOLS (https://zenodo.org/record/31628), and Map2Slim (http://search.cpan.org/~cmungall/go-perl/scripts/map2slim) exist to utilize GO annotations in enrichment analyses. These tools solve an essential task of “mapping” specific GO terms to more general GO terms by traversing appropriate edges in the GO graph structure. However, all current methods fail to utilize all the semantic information available in this ontology due to inconvenient features in the anatomy of GO.

### Anatomy of the gene ontology

The GO database represents a controlled vocabulary (CV) of biological and biochemical terms that are each assigned a unique alphanumeric code, which is used to annotate genes and gene products in many other databases, including UniProt [5] and Ensembl [6]. The ontology is divided into three sub-ontologies: Cellular Component (CC), Molecular Function (MF), and Biological Process (BP). Each can be envisioned as a graph or network where terms are nodes connected by edges, referred to as relations, that describe how each term relates to one another. For example, the term “connective tissue development” (GO:0061448) is connected to the term “tissue development” (GO:0009888) by the is_a relation. In this case, ontological terminology defines the term “tissue development” as a “parent” of the term “tissue development”. Likewise, “tissue development” (GO:0009888) is_a “anatomical structure development” (GO:0048856), which in turn is_a “developmental process” (GO:0032502). From a GO term mapping perspective, “connective tissue development” (GO:0061448) is_a “developmental process” (GO:0032502). The three sub-ontologies mentioned are “is_a disjoint” meaning that there are no is_a relations connecting any node among the three ontologies. However, other relations, such as “regulates,” connect nodes of separate sub-ontologies. Relations of interest to this study are part_of and has_part. These are like is_a in that they describe scope, i.e. relative generality or encompassment, but are separate in that is_a represents true sub-classing of terminology while part_of and has_part describe part-whole (mereological) correspondence. Therefore, we consider scoping relations to be comprised of is_a, part_of, and has_part, and mereological relations to be comprised of part_of and has_part.

There are three versions of the GO database, each containing aspects of the CV with varying complexity: *go-basic* is filtered to exclude relations that span across multiple sub-ontologies and to include only relations that point toward the root of the ontology; *go* or *go-core* contains additional relations, such as has_part that may span sub-ontologies and which point both toward and away from the root of the ontology; and *go-plus* contains yet more relations in addition to cross-references to entries in external databases like the Chemical Entities of Biological Interest (ChEBI) ontology [7]. The first and second versions are available in the Open Biomedical Ontology (OBO) flat text file formatting, while the third is available only in the Web Ontology Language (OWL) RDF/XML format.

### Path traversal issues in GO

Ontological graphs are typically designed as directed graphs, meaning that every edge has directionality, or directed acyclic graphs (DAGs), meaning that no path exists that leads back to a node already visited if one were to traverse the graph stepwise. This allows the graph to form a complex semantic model of biology containing both general concepts and more-specific (fine-grained) concepts. The “parent-child” relation hierarchy allows biological entities to be annotated at any level of specificity (granularity) with a single term code, as fine-grained terms intrinsically capture the meaning of every one of its parent and ancestor terms through the linking of relation-defining is_a edges in the graph. However, it is deceptively non-trivial to reverse the logic and organize similar fine-grained terms into general categories—such as those describing whole organelles or concepts like “DNA repair” and “kinase activity”—without significant manual intervention. This is due, in part, to the lack of explicit scoping, scaling, and other semantic correspondence classifiers in relations. Therefore, it is not readily clear how to classify terms connected by non-is_a relation edges. Although edges are directional, the semantic correspondence between terms connected by a scoping relation is computationally ambiguous, e.g. assessing whether term 1 is more/less general or equal in semantic scope with respect to term 2 is currently not possible without explicitly defining rules for such situations.

Ambiguity in assessing which term is more general in a pair of terms connected by a relation edge is confounded by the fact that edges describing mereological relations, such as part_of and has_part, are not strictly and universally inverse of one another. For instance, while every “nucleus” is part_of “cell,” not every “cell” has_part “nucleus.” Similarly, while every “nucleus” has_part “chromosome”, not every “chromosome” is part_of “nucleus” under all biological situations. Therefore, mereological edges are not necessarily reciprocal. Ontological logic rules, called axioms, ensure that this logic is maintained in the graph representation by allowing edges of the appropriate type to connect terms only if the inferred relation is universal [8]. GO maintains its own set of axioms regarding the relations it contains (http://www.geneontology.org/page/ontology-relations). This axiomatic representation is crucial to avoid making incorrect logical inferences regarding universality but does nothing to facilitate categorization of terms into parent concepts, especially since some mereological edges point away from the root of the ontology, toward a narrower scope. If these edges are followed, terms of more broad scope may be grouped into terms of more narrow scope, or worse, cycles may emerge which would abolish term hierarchy and make both categorization and semantic inference impossible. To circumvent this problem, some ontologies release versions that do not contain these types of edges. For GO, this is accomplished by go-basic. However, information is lost when these edges are removed from the graph. When attempting to organize fine-grained terms into common concepts using the hierarchical structure, this information loss can be significant because many specific-to-generic term mappings can utilize the same edge in many paths.

### Axiomatic versus semantic scoping interpretation of mereological relations in GO

While ensuring mereological universality in relation associations using current axioms is important within the purview of ontology development, for those interested in organizing datasets of gene annotations into relevant concepts for better interpretation—such is the case in annotation enrichment—it is important to utilize the full extent of the information within an ontology.

Current axiomatic representation of mereological relations requires the use of ontology versions which lack certain relations (http://geneontology.org/page/go-slim-and-subset-guide), resulting in a loss of retrievable information. If has_part edges—which point toward terms of narrower scope—were to be inversed to resemble part_of edges—ensuring that all edges point toward terms of a broader scope—terms could be effectively categorized with respect to semantic scope using the native graph hierarchy without losing any information in the process. However, this isn’t logically possible because of issues dealing with universality.

Therefore, we acknowledge the importance of existing axioms which prohibit reversing mereological edges in ontologies under the context of drawing *direct* semantic inferences. However, we maintain that in the context of detecting enriched broad concepts based on “summarizing” annotated fine-grained terms contained within differential annotation datasets, it is appropriate to evaluate mereological relations from a scoping perspective, which requires that all mereological edges point to their whole. This conundrum preventing the comprehensive categorization of GO terms can be dealt with by adding a single new relation to the ontology: part_of_some. Semantically, this relation deals with both the issue of universality and with the issue of the direction of granularity.

### GO Categorization Suite (GOcats)

For the issues stated above, we have developed a new tool called the GO Categorization Suite (GOcats). Fundamental to GOcats’ categorization algorithm is the re-evaluation of the has_part edge as part_of_some—correcting semantic correspondence inferences while ensuring ubiquitous use of all categorization-relevant relations in GO.

In comparing GOcats’ inclusion of re-evaluated has_part relations to the traditional method of ignoring has_part relations altogether and to the erroneous method of misinterpreting native has_part directionality, we illuminate the theoretical extent of information loss or potential for misinterpretation of has_part relations, respectively. Furthermore, in two independent enrichment analyses of real data—from a publicly available breast cancer dataset [9] and from samples investigating horse cartilage development [10], we demonstrate that GOcats’ reinterpretation of has_part can retain all information from GO while drawing appropriate categorical inferences in the context of annotation enrichment. Finally, we show that this reinterpretation has the added benefit of improving the statistical power of annotation enrichment analyses.

### Design and implementation

The *go-core* version of the GO database was chosen in favor of the *go-basic* version, because it contains the has_part edge relation which points away from the root of the ontology and because it contains other edges which connect the separate subontologies. Since one of our goals is to reinterpret mereological relations with respect to semantic scope, it is necessary that these relations be evaluated. Similarly, we excluded the *go-plus* version from this investigation, because we are not yet concerned with the reevaluation of the additional relations or database cross-references provided by *go-plus*.

**Fig 1:**
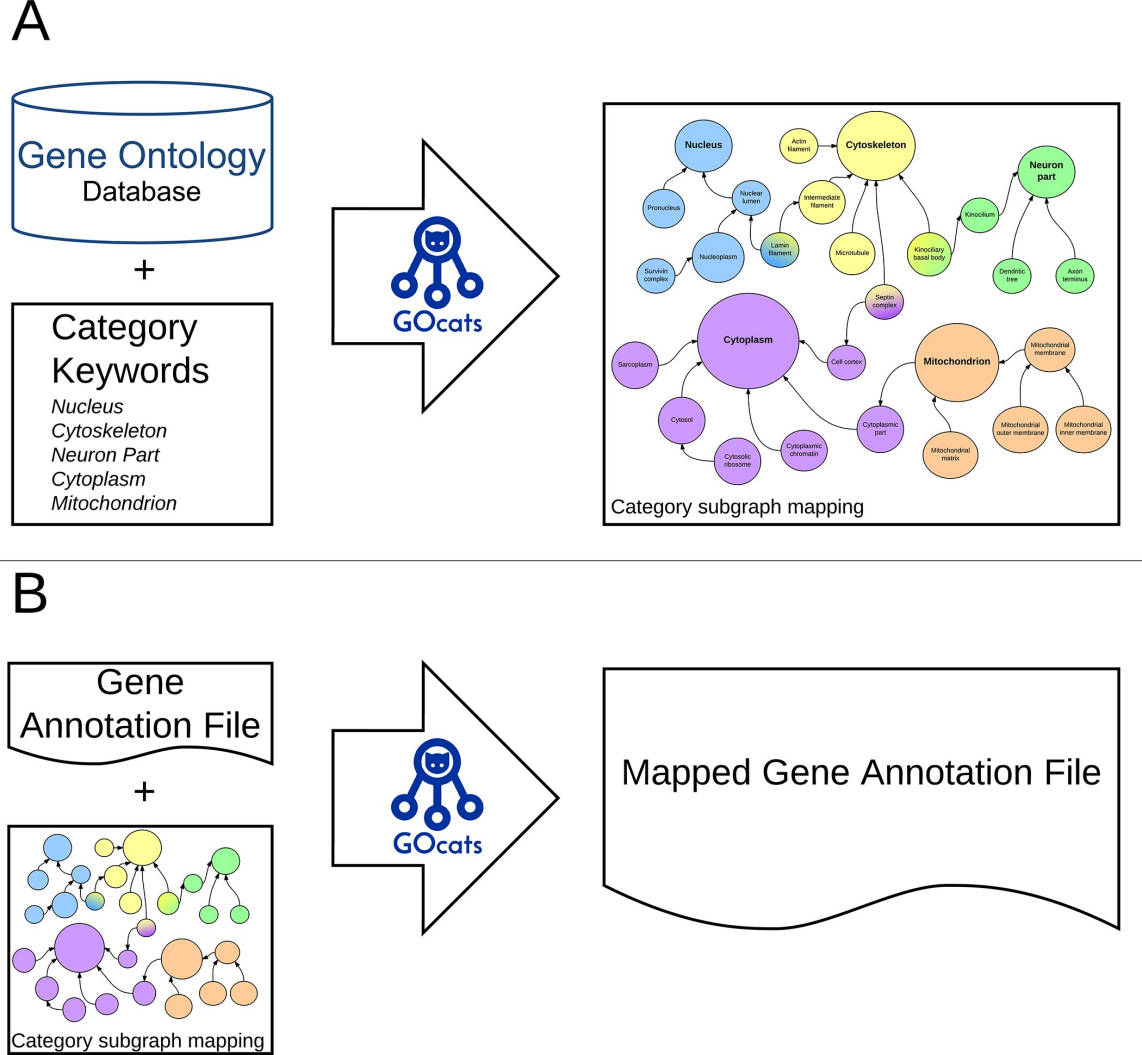
GOcats data flow diagram for creating categories of GO. A) GOcats enables the user to extract subgraphs of GO representing concepts as defined by keywords, each with a root (category-defining) node. B) Subgraphs extracted by GOcats are used to create a mapping from all sub-nodes in a set of subgraphs to their category-defining root node(s). This allows the user to map gene annotations in GAFs to any number of customized categories.

While *go-basic* is a true DAG, *go-core* is not strictly acyclic due to the additional has_part relations. However, when we inverse the traversal of has_part into the part_of_some interpretation, acyclicity is maintained. Therefore, we refer to our modified *go-core* graph as a DAG. GOcats is a Python package written in version 3.4.2 of the Python program language [11]. GOcats parses go-core and represents it as a DAG hierarchal structure. GOcats extracts subgraphs of the GO DAG (sub-DAGs) and identifies a representative node for each category in question (Fig 1). While GOcats’ categorization algorithms are a major feature of the software [12], it is not a focus of this study. Full API documentation for GOcats is available online (https://gocats.readthedocs.io).

**Fig 2:**
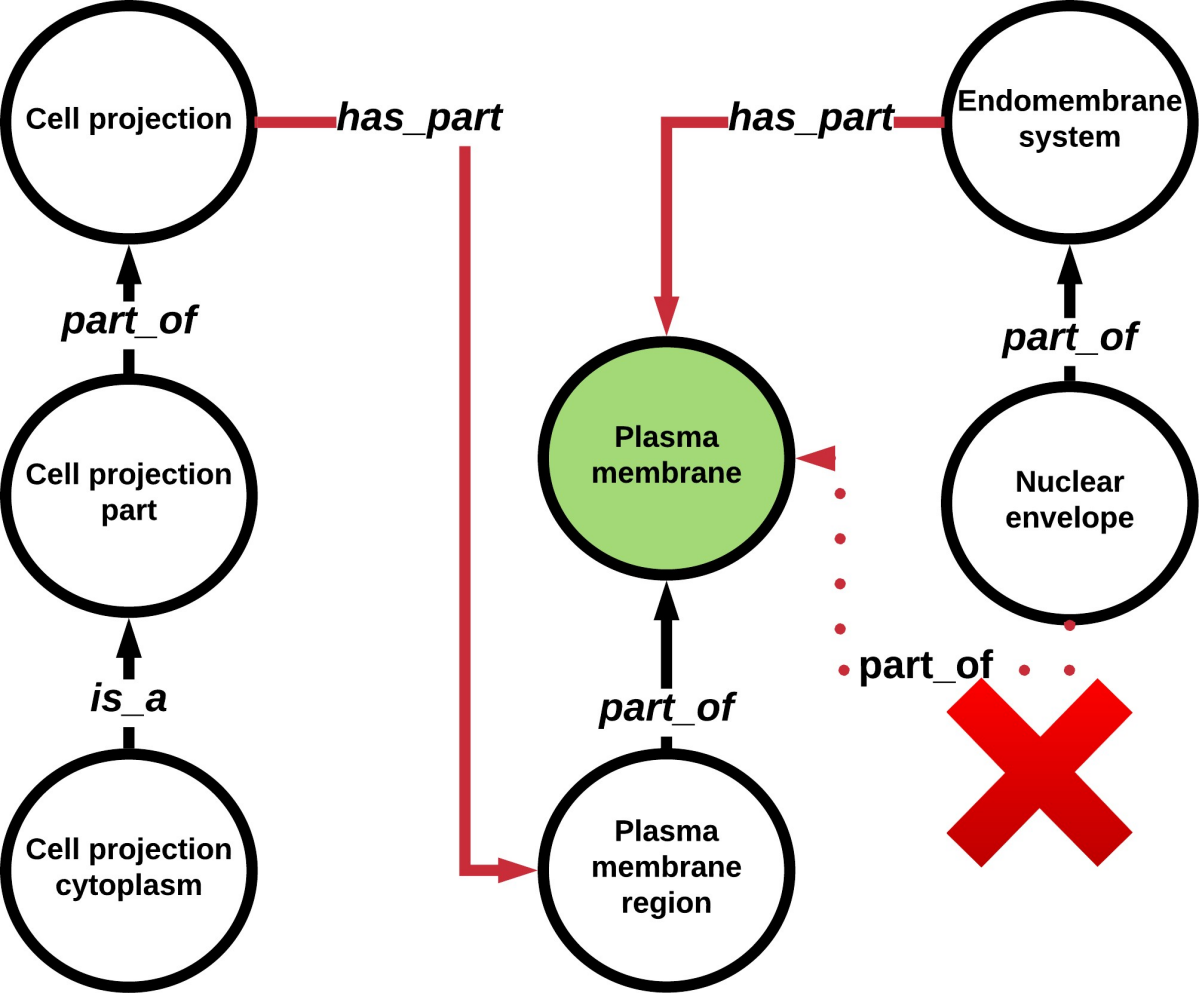
The has_part relation creates incongruent paths with respect to semantic scoping. Some tools may create questionable GO term mappings, i.e. “nuclear envelope” to “plasma membrane,” since the has_part relation edges point in from super-concepts to sub-concepts. GOCats avoids this by re-interpreting the has_part edges into part_of_some edges.

To overcome issues regarding scoping ambiguity among mereological relations, we hard-coded assigned properties indicating which term was broader in scope and which term was narrower in scope to each edge object created from each of the scope-relevant relations in GO. For example, in the node pair connected by a part_of or is_a edge, node 1 is narrower in scope than node 2. Conversely, node 1 is broader in scope than node 2 when connected by a has_part edge (Table 1, Fig 2). This edge is therefore reinterpreted by GOcats as part_of_some. While the default scoping relations in GOcats are is_a, part_of, and has_part, the user has the option to define the scoping relation set. For instance, one can create go-basic-like subgraphs from a go-core version ontology by limiting to only those relations contained in go-basic. For convenience, we have added a command line option, “go-basic-scoping,” which allows only nodes with is_a and part_of relations to be extracted from the graph.

**Table 1:** Frequency of relations in the gene ontology and suggested semantic correspondence classes to reduce ambiguity†.

Relationship	Frequency in GO (CC+BP+MF)	Frequency in GO CC	Frequency in GO BP	Frequency in GO MF	Correspondence Class	Correspondence Members
is_a	72455	5591	54689	12175	Scoping (hyponymy)	hyponym "is_a" hypernym
part_of	8613	1702	5751	1160	Scaling (meronymy)	meronym "part_of" holonym
has_part	736	156	339	241	Scaling (meronymy)	holonym "has_part" meronym
happens_during	24	0	24	0	Spatiotemporal (process-process)	process "happens_during" process
ends_during	1	0	1	0	Spatiotemporal (process-process)	process "ends_during" process
occurs_in	181	0	180	1	Spatiotemporal (process-entity or process-process)	process "occurs_in" entity OR process "occurs_in" process
regulates	3368	0	3322	46	Active (actor-subject)	actor "regulates" subject
positively_regulates	2916	0	2880	36	Active (actor-subject)	actor "positively_regulates" subject
negatively_regulates	2937	0	2285	52	Active (actor-subject)	actor "negatively_regulates" subject
regulated_by^‡^	0	0	0	0	Active (actor-subject)	subject "regulated_by" actor
before^‡^	0	0	0	0	Spatiotemporal (prior-latter)	prior "before" latter

† GO-core data-version: releases/2016-01-12 (available in Scripts Directory)

‡ These relationships are not found in GO but are part of the Relations Ontology

## Results

### GOcats’ reinterpretation of the has_part relation increases the information retrieval from GO and avoids potential misinterpretations of ambiguous relationship inferences

GOcats reevaluates path tracing for the has_part edge to make it congruent with other relations that delineate scope. With path tracing unchanged, has_part edges lead to erroneous term mappings unless they are completely excluded from the ontology. To evaluate the extent of incorrect semantic interpretation conferred by has_part relations, we calculated all potential false mappings (pM_F_) between nodes for a given GO sub-ontology by counting the number of mappings from all children of a has_part edge to all parents of a has_part edge assuming the original GO has_part edge directionality. Next, we compared the pM_F_ to the total number of true mappings (M_T_) for a given GO sub-ontology to evaluate the possible magnitude of their impact (Methods, Eqs 1–5, Scripts Directory 1,2). As shown in Table 2, there are 23,640 pM_F_s in Cellular Component, 8,328 pM_F_s in Molecular Function, and 89,815 pM_F_s in Biological Process. Comparatively, the amount of pM_F_s is 42%, 13%, and 16% the size of the M_T_, in Cellular Component, Molecular Function, and Biological Process, respectively.

**Table 2:** Prevalence of potential has_part relation mapping errors in GO.

Sub-Ontology	Estimated Potential False Mappings (epM_F_)	True Mappings (M_T_)	M_T_ ∩ epM_F_	Potential False Mappings pM_F_ = epM_F_—(M_T_ ∩ epM_F_)	True Mappings without HP (_IA_PO_M_T_)*	Lost Mappings (M_T_—_IA_PO_M_T_)*
Cellular Component	30036	56025	6396	23640	49679	6346
Molecular Function	10074	62436	1746	8328	56194	6242
Biological Process	93092	555543	3277	89815	527869	27674

* IA_PO refers to a graph created with only is a and part of relationship edges.

The conventional solution to avoid these errors is to use versions of ontologies that remove edges like has_part. [13]. Considering the number of possible mappings between terms as a measure of information content, we quantified the loss of information acquired when has_part is omitted during mapping by subtracting the number of M_T_ in graphs containing is_a, part_of, and has_part edges from those with only is_a and part_of edges. As shown in Table 2, Cellular Component lost 6,346 mappings, Molecular Function lost 6,242 mappings, and Biological Process lost 27,674 mappings, which equates to 11%, 10%, and 5% loss of information in these sub-ontologies, respectively. It is important to note here that the mapping combinations were limited to those nodes containing is_a, part_of, and has_part relations only. Because paths in GO are heterogeneous with respect to relation edges, this loss of information is a lower-bound estimate since other relations exist that connect additional nodes, but in a manner unusable for semantic correspondence interpretation. This is especially true for Biological Process, which has many regulatory relations that were not evaluated here.

While the potential for false mappings are high considering the has_part relation alone, this statistic does not illuminate the scale of the issue facing users of current ontology mapping software. Importantly, it does not address a fundamental limitation and danger facing software like map2slim (M2S) (http://search.cpan.org/~cmungall/go-perl/scripts/map2slim), which non-discriminately evaluates relation edges. For example, terms linked by an active relation like *regulates*, or by the has_part edge are categorized as if they are related by a scoping relation like is_a. Therefore, we calculated the total number of possible mappings produced by M2S and enumerated the intersection of these mappings against those made by GOcats which were constrained to paths that contained only scoping relations, is_a, part_of, and has_part (Methods, Eqs 6 and 7). Overall, M2S made 325,180 GO term mappings, i.e. categorizations, which did not intersect GOcats’ full set of corrected scoping relation mappings. We consider these false mapping pairs (M_pair,M2S_), since they represent a problematic evaluation of scoping semantics. This contrasted with 710,961 correct mappings that intersected the GOcats mapping pairs (M_pair,GOcats_) giving a percent error of 31.4%. Cellular Component, Molecular Function, and Biological Process contained 22,059, 29,955 and 273,166 erroneous mappings, which accounted for respective percent errors of 30.7%, 34.8%, and 31.1% (Table 3).

**Table 3:** Summary of GO term mapping errors resulting from misevaluation of relations with respect to semantic scoping.

(Sub) Ontology	Map2Slim Mappings (M_pair,M2S_ont_)*	GOcats Scoping Mappings (M_pair,Gocats_ont_)*	Potentially false Map2Slim Mappings pM_F,M2S_ = M_pair,M2S_ - (M_pair,M2S_ ∩ M_pair,Gocats_all_)*	Map2Slim Correct Mappings M_T,M2S_ = M_pair,M2S_ ∩ M_pair,Gocats_all_*	Possible Map2Slim Error Fraction pM_F,M2S_ / M_pair,M2S_ont_
All GO	1036141	820467	325180	710961	0.314
Cellular Component	71835	56025	22059	49776	0.307
Molecular Function	86163	62436	29955	56208	0.348
Biological Process	878143	555543	273166	604977	0.311

* GOcats_all refers to GOcats-derived mapping pairs across all of GO, while GOcats_ont refers to GOcats-derived mapping pairs for the indicated ontology in each row.

### GOcats’ reinterpretation of has_part relations provides improved annotation enrichment statistical power

We incorporated GOcats-derived ontology ancestor paths (paths from fine-grained terms to more general, categorical terms) into the categoryCompare version 1.99.158 [14] annotation enrichment analysis pipeline and performed annotation enrichment on an Affymetrix microarray dataset of ER+ breast cancer cells with and without estrogen exposure [9]. We compared these enrichment results to those produced when unaltered ancestor paths from GO—excluding the has_part relation—were incorporated into the same categoryCompare pipeline (Methods, Scripts Directory 3*)*.

We also performed enrichment analyses comparing the ancestor traversals of DEseq2 differential gene expression datasets across time points during the fetal development of two cartilage tissue types in *Equus caballus* (Methods, Scripts Directory 4*)*.

**Fig 3:**
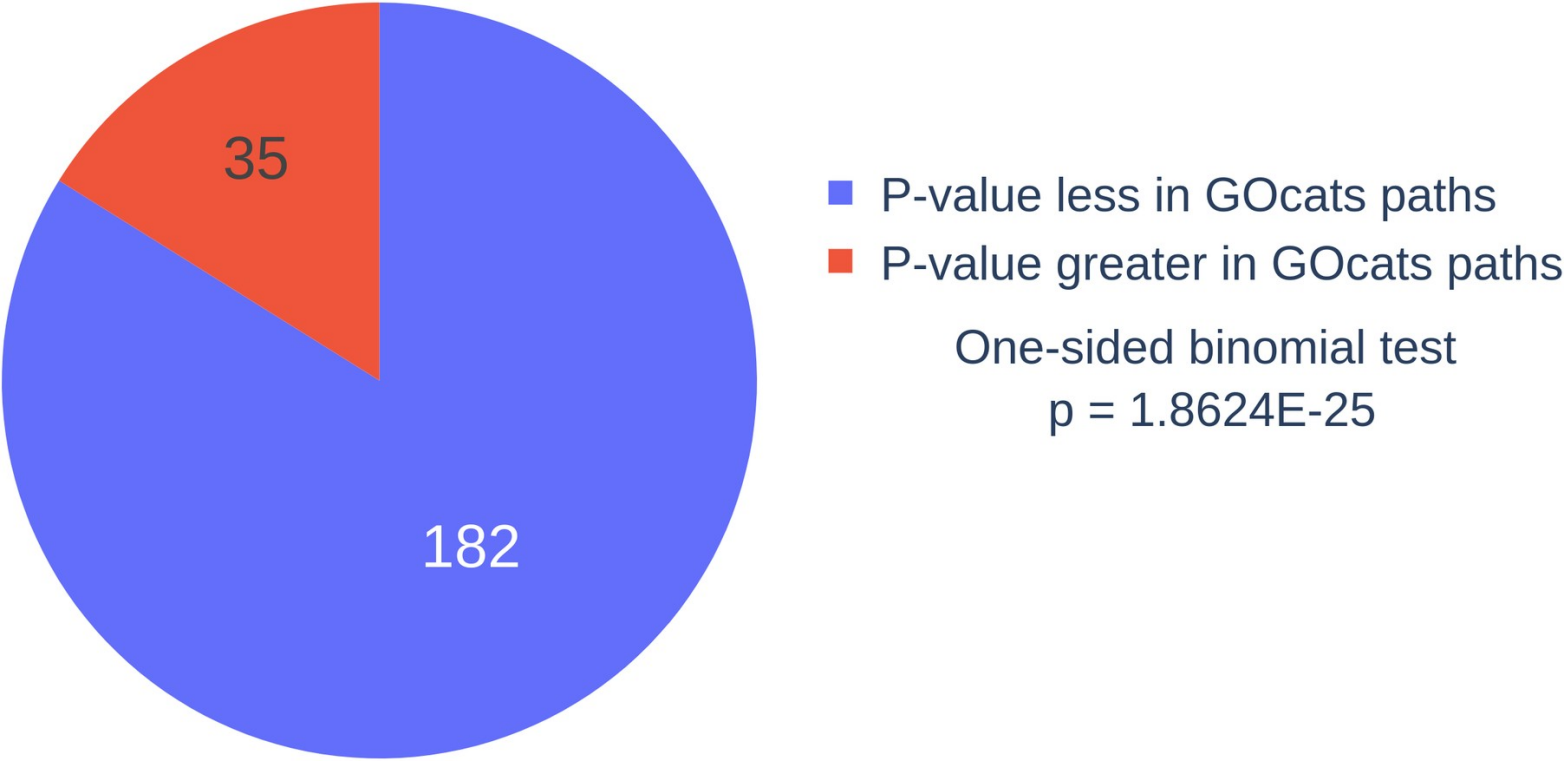
Comparison of adjusted p-values for significantly-enriched annotations using GOcats paths vs excluding has_part edges. Most significantly-enriched GO terms had an improved p-value when GOcats re-evaluated has_part edges for the enrichment of the breast cancer data set in this investigation.

Assessment of adjusted p-values from significantly enriched terms using GOcats’ paths versus the traditional method that omits has_part edges shows that GOcats reliably improves the statistical significance of term enrichment results through its re-interpretation of *has_part* relation semantics (Fig 3 and Table A in S1 File). In the breast cancer dataset, of the 217 significantly enriched terms found using the traditional enrichment method at an alpha of 0.01 for FDR-adjusted p-values, 182 had adjusted p-values that were improved when GOcats part_of_some paths were used. This number of improved p-values is statistically significant as indicated by a one-sided binomial test p-value of 1.86E-25 (i.e. 1.86 x 10^−25^).

Additionally, GOcats was able to identify 15 unique significantly-enriched terms at an alpha of 0.01 for adjusted p-values that would otherwise be omitted due to the loss of has_part edges (Table 4). Four of these terms involve purinergic nucleotide receptor activity, which has been implicated elsewhere in other investigations related to breast cancer in both ER+ and ER- breast cancer cell lines [15].

**Table 4:** Uniquely enriched terms between GOcats paths and traditional paths from the breast cancer dataset analysis.

GO Term	Description	Adjusted p-value	Uniquely enriched in
GO:0035590	purinergic nucleotide receptor signaling pathway	0.000119296	GOcats
GO:0016502	nucleotide receptor activity	0.000103448	GOcats
GO:0035586	purinergic receptor activity	0.000129432	GOcats
GO:0036387	pre-replicative complex	6.03E-05	GOcats
GO:0042023	DNA endoreduplication	2.70E-10	GOcats
GO:0006313	transposition, DNA-mediated	1.31E-28	GOcats
GO:0031261	DNA replication preinitiation complex	5.55E-06	GOcats
GO:0032196	transposition	1.31E-28	GOcats
GO:0004888	transmembrane signaling receptor activity	0.006197782	GOcats
GO:0035587	purinergic receptor signaling pathway	0.000129432	GOcats
GO:0098039	replicative transposition, DNA-mediated	1.31E-28	GOcats
GO:0099600	transmembrane receptor activity	0.006197782	GOcats
GO:0001614	purinergic nucleotide receptor activity	0.000119296	GOcats
GO:0005656	nuclear pre-replicative complex	6.03E-05	GOcats
GO:0000988	transcription factor activity, protein binding	0.002944403	GOcats
GO:0051716	cellular response to stimulus	0.008043537	Traditional paths
GO:0007059	chromosome segregation	1.54E-06	Traditional paths
GO:0045005	DNA-dependent DNA replication maintenance of fidelity	0.001514676	Traditional paths
GO:0008094	DNA-dependent ATPase activity	0.000454406	Traditional paths
GO:0140097	catalytic activity, acting on DNA	6.04E-09	Traditional paths
GO:0050896	response to stimulus	0.000712619	Traditional paths
GO:1902969	mitotic DNA replication	0.001852706	Traditional paths

GOcats’ path tracing showed similar improvements when comparing p-values from GO annotation enrichment derived from the differential gene expression analyses between horse cartilage development time points (Table 5). In this analysis (see Methods), neighboring time point analyses (early and late) were compared to extreme time point analyses (extreme) (Table 6). The traditional enrichment method yielded between 82 to 233 total enriched terms, with 67% to 92% of these terms’ adjusted p-values being improved when GOcats ancestor path tracing was used. Quantifying the improvements in the p-values via a binomial test generates p-values ranging from 1.32E-03 to 2.58E-44 (i.e 1.32 x 10^−3^ to 2.58 x 10^−44^). Even with a Bonferroni multiple test correction, the adjusted p-value of the six binomial tests performed range from 7.92E-03 and 1.55E-43.

**Table 5:** Binomial test results for GOcats vs no_hp enrichment for horse cartilage development time point comparisons.

Tissue Type	Time Series Comparison	Total Enriched Terms	Enriched Terms with Lower P-value with GOcats*	One-sided Binomial Test
Anlagen	45-day fetal to 60-day fetal (early)	228	183	6.22E-21
	60-day fetal to neonatal (late)	140	129	5.31E-27
	45-day fetal to neonatal (extreme)	158	139	5.01E-24
Interzone	45-day fetal to 60-day fetal (early)	82	55	1.32E-03
	60 day fetal to neonatal (late)	233	196	1.23E-27
	45-day fetal to neonatal (extreme)	233	215	2.58E-44

*The enriched terms with improved adjusted p-values from GOcats traversal.

**Table 6:** Neighbor vs extreme time point comparison of enriched terms in horse cartilage development enrichment analyses.

Tissue type	GO Term Set	Terms in set
anlagen	EarlyEnrichedTerms	50
	EarlySupportedEnrichedTerms^**ⱡ**^	1
	EarlyUniqueEnrichedTerms_Gocats_^**ⱡ**^	49
	LateEnrichedTerms	41
	LateSupportedEnrichedTerms^**ⱡ**^	0
	LateUniqueEnrichedTerms_Gocats_^**ⱡ**^	41
Interzone	EarlyEnrichedTerms	22
	EarlySupportedEnrichedTerms^**ⱡ**^	3
	EarlyUniqueEnrichedTerms_Gocats_^**ⱡ**^	19
	LateEnrichedTerms	81
	LateSupportedEnrichedTerms^**ⱡ**^	3
	LateUniqueEnrichedTerms_Gocats_^**ⱡ**^	78

ⱡ Sets defined in Eqs 8–11

Also, all but one of the binomial test p-values was below 6.22E-21; however, the comparison of the fetal interzone tissue at 45 days of gestation to neonatal epiphyseal cartilage had drastically fewer total enriched terms. Furthermore, GOcats was able to identify additional significantly-enriched terms from the first and second neighboring time point analyses as compared to the traditional method applied to the extreme analysis. GOcats extracts a notable number of uniquely enriched terms from the individual time point comparisons (Table 6, UniqueEnrichedTerms_GOcats_). A few of these enriched terms (Table 6, SupportedEnrichedTerms) are directly supported by the traditional method enrichment of the extreme time point comparisons. In other words, the traditional method enrichment of the extreme time point comparisons provides some ground truth for validating uniquely enriched terms detected by the GOcats enrichment analysis of the nearest-neighbor time point comparisons.

## Discussion

### Issues with semantic correspondence

As early as the late 1980s, explicit definitions of semantic correspondence for a relation between ontological terms have been stressed in the context of relational database design [16]. This includes concepts of part-whole (mereology), general-specific (hyponymy), feature-event, time-space (i.e spaciotemporal relations), and others. OBO’s and GO’s ontological edges are directional insofar as their relations accurately describe how the first node relates to the second node empirically, providing axioms for deriving direct semantic inferences. However, the directionality of these edges is ambiguous in that they do not explicitly describe how the terms relate to one another semantically in terms of scope, and this is due largely to the lack of explicit semantic correspondence qualifiers.

A simple way to avoid mapping problems associated with non-scoping relation direction is to omit those relations from the analysis. This strategy avoids incorrect scoping interpretation at the expense of losing information. As an example, EMBL-EBI’s QuickGO term mapping service omits has_part type under its “filter annotations” by GO identifier options [13]. Furthermore, Bioconductor’s GO.db (https://bioc.ism.ac.jp/packages/3.3/data/annotation/html/GO.db.html) also avoids mapping issues by indirectly omitting this relation; it uses a legacy MySQL dump version of GO which does not contain relation tables for has_part. We argue that while avoiding problematic relations altogether does prevent scope-specific mapping errors, it also limits the amount of information that can be gleaned from the ontology. By eliminating has_part from graphs created by GOcats, we see a ~11% decrease in information content (as indicated by a decrease in the number possible mappings) in Cellular Component. Likewise, there is a 10% and 5% decrease of information content in Molecular Function and Biological Process, respectively (Table 2). Thus, omitting these relations from analyses removes a non-trivial amount of information that could be available for better interpretation of functional enrichment. However, the total impact is not completely appreciated here, because not all relations were evaluated in this study; only the scoping relations of is_a, part_of, and has_part. The potential for additional information loss is very high in Biological Process, for example, when considering the large number of unaccounted relations: regulates, positively_regulates, and negatively_regulates (Table 1). These relations add critical additional regulatory information to ontological graph paths, which would also be lost when ignoring the has_part relation, if they occurred along a path that also contained has_part. The same is also true for Molecular Function, although the frequency of additional, non-scoping relations are lower.

Furthermore, automated summarization of annotations enriched in gene sets requires a more sophisticated evaluation of the scoping semantics contained in ontologies, which prior tools are not fully equipped to provide. M2S is one widely-utilized GO term categorization method that is available as part of the OWLTools Java application (https://github.com/owlcollab/owltools). The Perl version of M2S has been integrated into the Blast2GO suite since 2008 [17] and this gene function annotation tool has been cited in over 1500 peer-reviewed research articles (Google Scholar as of Nov. 28, 2017). We verified that the Perl and Java versions of M2S produced identical GO term mappings for a given dataset and GO slim, and therefore have the same mapping errors (Scripts Directory 2). Although the number of pM_F_s reported in the results represent the upper limit of the possible erroneous mappings, the fact that at least 120,000 of these exist in GO for the has_part relation alone or that the removal of this edge type results in up to an 11% reduction of information content provide bounds on the scope of the issue. To be clear, tools like M2S can be safe and not produce flawed mappings if they are used alongside ontologies that contain only those relations that are appropriate for evaluation, such as go-basic. However, we intentionally utilized *go-core* to illustrate the danger in using tools that do not provide explicit semantic control on how ontologies are utilized.

GOcats represents a step toward a more thorough evaluation of the semantics contained within ontologies by handling relations differently according to the type of correspondence that they represent. In the case of relations such as has_part, this involves altering the correspondence directionality for the task at hand, which is to organize terms into categories. As a proof-of-concept, we classified the is_a, has_part, and part_of relations into a common “scoping” correspondence type and hard-coded assigned graph path tracing heuristics to ensure that they are all followed from the narrower-scope term to the broader-scope term. One caveat of this approach is that because of previously mentioned issues in universality logic, the inverse of has_part is not strictly part_of, but rather part_of_some. We argue that the highly unlikely misinterpretation of universality in this strategy is preferable to the loss of information experienced when using trimmed versions of ontologies for term categorization. To elaborate, most current situations calling for term categorization involve gene enrichment analyses. Spurious incorrect mappings through part_of_some edges would not enrich to statistical significance, unless a systematic error or bias is present in the annotations. Even if a hypothetical term categorization resulted in enrichment of a general concept that was not relevant to the system in question (i.e. “nucleus” enriched in a prokaryotic system), it would be relatively straight-forward to reject such an assignment by manual curation and find the next most relevant term. Conversely, it is not reasonable to manually curate all possible missed term mappings resulting from the absence of an edge type in the ontology.

Another potential complication in semantic correspondence of relations is that some relations are *inherently* ambiguous. The clearest example of this again can be found in the well-utilized part_of relation. This relation is used to describe relations between physical entities and concepts (e.g. “nuclear envelope” part_of “endomembrane system”) and between two concepts (e.g. “exit from mitosis” part_of “mitotic nuclear division”) with no explicit distinction. To address the former issue, future work will augment our use of hard-coded categorization of semantic correspondences through the development of heuristic methods that identify and categorize these among the hundreds of relations in the Relations Ontology (http://www.obofoundry.org/ontology/ro.html) [18]. As a good starting point, we suggest using five general categories of relational correspondence for reducing ambiguity (Table 1): scope (hyponym-hypernym), mereological, a subclass of scope (meronym-holonym), spatiotemporal (process-process, process-entity, entity-entity), active (actor-subject), and other.

### Using GOcats for annotation enrichment

While we reported the loss of information available for annotation enrichment with has_part excluded from GO and quantified the effect of incorrect inferences that can be made if has_part is included in GO during enrichment, these results only represent hypothetical effects that might be overcome when GOcats reinterprets this relation. One of GOcats’ original intended purposes was to improve the interpretation of results from annotation enrichment analyses. However, in the process of designing heuristics to appropriately categorize GO terminology, we also sought to overcome the limitations that come with following the traditional methods of path tracing along relations in GO. Here we focused on overcoming the loss of information encountered when ignoring has_part relations. Our solution was to re-evaluate these relations under the logic of part_of_some and invert the direction of has_part. While this re-interpretation is limited in usage, we believe that, in the scope of annotation enrichment, it is valid for reasons previously explained.

Our first evaluation of enrichment results compared GOcats ancestor paths to traditional GO ancestor paths in the enrichment analysis of an older, publicly-available microarray breast cancer dataset, generated from an Affymetric HG-U95Av2 array which only covered 9000 genes. With this comparison, we demonstrate a highly statistically significant improvement (p-value = 1.86E-25) in the statistical power of annotation enrichment analysis. Specifically, 182 out of 217 significantly enriched GO terms from the traditional analysis had improved p-values in the GOcats-enhance enrichment analysis. Importantly, we also detect significantly enriched GO terms in the GOcats’ results that were not detected using the traditional analysis. The inclusion of the re-interpretation of has_part edges allowed for the significant enrichment (adjusted-p-value < 0.002 with FDR set to 0.01) of four terms related to purinergic nucleotide receptor signaling which has been associated with ER+ MCF-7 breast cancer cell proliferation [19,20]. Furthermore, purinergic nucleotide receptor signaling has been implicated in predicting breast cancer metastasis in other studies; however, these studies involved ER- metastatic breast cancer cell lines [21]. We again confirmed this effect in our evaluation of GO annotation enrichment results of recently collected, RNAseq horse cartilage development datasets. Here we saw an improvement in 67% to 92% of enriched terms across the six time point enrichment analyses. Fundamentally, the addition of part_of_some interpretation of has_part relations improves the statistical power of the annotation enrichment analysis, allowing the detection of additional enriched annotations with statistical significance from the same dataset. In addition, the GOcats annotation enrichment analysis extracts a notable number of uniquely enriched annotations from the neighboring, individual time point differential gene expression analyses. Some of these uniquely enriched terms are directly supported by the traditional annotation enrichment analysis of the extreme time point differential gene expression analyses (Table 6). These results on multiple datasets involving two separate experimental designs using both older and newer transcriptomics technologies demonstrate the ability of utilizing GOcats-augmented ontology paths to derive additional information from annotation enrichment analyses. While these results demonstrate an improvement in statistical power of annotation enrichment analyses, no data analysis method can address unknown bias in a dataset. Bias that leads to confounding factors is best addressed at the point of experimental design, but sometimes the effects from identified confounding factors can be mitigated after the experiment is performed during data analysis [22].

To conclude, GOcats enables the simultaneous extraction and categorization of gene and gene product annotations from GO-utilizing knowledgebases in a manner that respects the semantic scope of relations between GO terms. It also allows the end-user to organize ontologies into user-defined biologically-meaningful concepts—a feature that we have explained elsewhere [12]. This categorization lowers the bar for extracting useful information from exponentially growing scientific knowledgebases and repositories in a semantically safer manner. In summary, GOcats is a versatile software tool applicable to data mining, annotation enrichment analyses, ontology quality control, and knowledgebase-level evaluation and curation.

## Materials and methods

### Evaluating hypothetical false mapping and true mapping pairs in GO involving the has part relation

To determine how significant mapping issues are because of semantic scope inconsistencies with has_part relations, we built the GO graph, data-version: releases/2016-01-12 using only the scoping relations is_a, part_of, and has_part edges, while omitting other relation edges in the graph, such as regulates, happens_during, and ends_during. Next, we counted the number of potential false mappings (pM_F_) that could result if has_part was left in its unaltered directionality; i.e. the edge directionality that currently exists in GO. To accomplish this, we define sets of potentially problematic ancestors (PA_e_) for every has_part edge (e) as

$${PA}_{e}=\{{Ae}_{child}+e_{child}\}-\{{Ae}_{par}+e_{par}\}$$ (1)

where Ae_child_ and Ae_par_ are sets of nodes that are ancestors of the edge’s child and parent nodes, respectively, and e_child_ and e_par_ are the edge’s parent and child nodes. Similarly, we define the potentially problematic descendants (PD_e_) for every has_part edge (e) as

$${PD}_{e}=\{De_{par}+e_{par}\}-\{{De}_{child}+e_{child}\}$$ (2)

where De_par_ and De_child_ are sets of nodes that are descendants of the edge’s parent and child nodes, respectively. We then calculate the potential mappings that can occur across each edge, e by the following:

$${pM}_{F,e}=\{(d,a)|d\in {PD}_{e};a\in {PA}_{e}\}$$ (3)

The total number of potential false mappings that can result from an edge type, in this case the has_part relation, is given by

$$pM_{F}=|{⋃}_{e=1}^{n}pM_{F,e}|$$ (4)

Finally, we calculate the number of total possible true mappings (M_T_) between any two arbitrary nodes (n_1_, n_2_) in a given sub-ontology graph (G) in GO:

$$M_{T}=|\left\{n_{1}anc\cap n_{2}desc|n_{1}\in G;\;n_{2}\in G\right\}|$$ (5)

In Eq 6, we used GOcats to calculate the possible number of true mappings while considering is_a, part_of, and re-evaluated has_part (part_of_some) relations in GO.

### Evaluating hypothetical false mappings encountered when the unaltered has_part relation is parsed with Map2Slim

The Java implementation of OWLTools’ Map2Slim (M2S) does not include the ability to output a mapping file between fine-grained GO terms and their GO slim mapping target from the GAF that is mapped. To identify target ancestor terms of individual GO terms, we created a special custom GAF where the gene ID column and GO term annotation column of each line were each replaced by a different GO term for each GO term in Cellular Component, data-version: releases/2016-01-12. We then allowed M2S to map this GAF with a provided GO slim. The resulting mapped GAF was parsed to create a standalone mapping between the terms from the GO slim and a set of the terms in their subgraphs. Because M2S’s custom term list option removes terms subsumed by other mappings, we were forced to also perform separate mappings for each GO term; e.g. the entire GO was mapped to one GO term at a time for each ~44,000 terms. These computations were done in parallel on a small TORQUE-managed Linux cluster to complete the calculations in a reasonable amount of time. We combined and converted the results into a set of ordered term pairs (M_pair,M2S_), where the first position is the mapped term and the second position is the term to which the first is mapped; self-mappings were ignored. Using the GOcats’ evaluation of the three scoping relations, is_a, part_of, and has_part, to create the “correct” set of mappings in a scoping paradigm, we defined the set of potentially false M2S mappings (pM_f,M2S_) as

$$pM_{f,M2S}=\{M_{pair,M2S}\}-(\left\{M_{pair,M2S}\}\cap \{M_{pair,GOcats(scoping)}\right\})$$ (6)

where M_pair,GOcats(scoping)_ is the set of ordered GO term mapping pairs produced from GOcats, under the constraint that only scoping relations were used in the graph (is_a, has_part, and part_of). The ratio of potential false scoping-type mappings to correct scoping mappings produced by M2S (M2S_error_) is given by

$${M2S}_{error}=\frac{\left|pM_{f,M2S}\right|}{\left|\left\{M_{pair,GOcats(scoping)}\right\}\right|}$$ (7)

To look specifically at individual sub-ontologies, we filtered the M2S mapping pairs to those where both terms were a member of each sub-ontology. These were also intersected with the full set of GOcats mapping pairs. Scripts for generating these results can be found in Scripts Directory 1.

### Comparing mapping functionality between the Java and Perl versions of Map2Slim

To ensure that the same mapping errors encountered using the Java version of M2S, which is integrated in OWLTools, are also present in the Perl version of M2S (http://search.cpan.org/~cmungall/go-perl/scripts/map2slim), which is integrated in Blast2GO, we tested whether the mapping functionality was consistent between the two versions. Since the Perl version only supports GO slims and does not support custom specification of a list of GO terms, we compared the output of each version’s mapping of the HPA-sourced knowledge data to the “generic” GO slim dataset (http://geneontology.org/page/go-slim-and-subset-guide). Since some minor GAF formatting differences exist between the output files, we wrote a script to directly compare the gene-to-GO annotation mappings made by each version (Scripts Directory 2).

### Annotation enrichment analysis of breast cancer dataset

To evaluate the effects that GOcats ancestor paths had on real data, we performed GO annotation enrichment using categoryCompare [14]—and an updated version of the GO graph, data-version: releases/2017-12-02—on an Affymetrix microarray dataset of ER+ breast cancer cells with and without estrogen exposure [9]. In this dataset, we ignored time point information and only considered data associated with the presence and absence of estrogen exposure.

The categoryCompare package can consider GO ancestor terms for annotated terms in the experimental dataset when calculating enrichment. We therefore created two mapping dictionaries in Python where a key of each term in GO maps to a set of its ancestor terms in the GO graph. For the traditional method of inferring ancestors, we created this mapping from a version of the GO graph with the has_part relation omitted. For testing GOcats’ effect on enrichment, we created a version of this mapping with the has_part relation re-interpreted as part_of_some. We applied these ancestor mappings to all annotations in the human GOA database, generated: 2017-11-21 08:07 [23]. R scripts and Python scripts for generating the enrichment results can be found in Scripts Directory 3.

To compare FDR-adjusted (target FDR = 0.01) p-values between enrichment results produced by GOcats ancestors and traditional ancestors, we filtered the enriched terms identified by the traditional method with an alpha cutoff of 0.01 and counted the number of terms identified by GOcats’ analysis whose adjusted p-value was less than the traditional analysis. Identical adjusted p-values were ignored. We then performed a one-sided binomial test (i.e. “coin-toss analysis” with directional change from 0.5) comparing the number of significantly enriched adjusted p-values that improved with GOcats versus total number of enriched terms found in the traditional analysis (with identical adjusted p-values excluded). To identify uniquely enriched terms found using the GOcats-enhanced enrichment analysis, we compared the sets of significantly enriched terms (alpha cutoff 0.01 for adjusted p-values) in each enrichment results table and selected terms only found in the GOcats-enhanced set.

### Annotation enrichment analysis of horse cartilage development dataset

To further test the effects that GOcats’ ancestor path tracing has on term enrichment, we again performed GO annotation enrichment using categoryCompare [14] applied to differentially-expressed genes identified by DESeq2 from RNAseq datasets derived from developing equine cartilaginous tissues (interzone and anlagen) across two gestational time points and their neonatal derivatives (articular cartilage and epiphyseal cartilage, respectively). The time points were fetal interzone tissue at 45 days of gestation (iz_45); fetal anlagen tissue at 45 days (anl_45); fetal interzone tissue at 60 days of gestation (iz_60); anlagen fetal tissue at 60 days (anl_60); neonatal articular cartilage (ac_neo); and neonatal epiphyseal cartilage (epi_neo). At least six biological replicates were acquired for each tissue type and time point (separate horse fetuses from similar breeds) with RNA-seq readings of 30–40 million reads per sample.

We downloaded horse gene annotations from AgBase [24] and built two full ancestor annotation mappings for each gene, one using GOcats’ re-evaluation of the has_part relation and the other using the traditional method of omitting the has_part relation altogether.

For each pairwise time point comparison from the DESeq2 analyses (IZ/ANL_45-IZ/ANL_60, IZ/ANL_60-AC/Epi_neo, or IZ/ANL_45-AC/Epi_neo), we selected positively- or negatively-changing genes by filtering to those changing genes which had an adjusted p-value ≤ 0.01. Based on the sign of each gene’s fold expression from the dataset we classified these genes into categories for categoryCompare as “positive”, “negative”, or “all” (either positively or negatively changing in expression). Enrichment was performed on each of these three categories for each three pairwise time point comparisons (early, late, and extreme) for each two tissue types using two ancestor mappings: GOcats’ and the traditional omission of has_part, yielding 36 total enrichment analyses.

Using the enrichment results from the “all” category for each pairwise time point comparison and tissue type, we again evaluated the improvement in the adjusted p-value seen using the GOcats’ ancestors when compared to the traditional method of mapping ancestors using a binomial test (see *Annotation enrichment analysis of breast cancer dataset*).

In addition to the “positive”, “negative”, and “all” gene sets identified from the individual pairwise time point analyses, we also defined special gene sets relating to the scope of the whole time series. These were defined as i) early: those genes that significantly increased or decreased in fold-change during the iz/anl_45-iz/anl_60 time point comparison but did not significantly change in the iz/anl_60-ac/epi_neo time point comparison, ii) late: those genes that did not have a significant fold-change in the iz/anl_45-iz/anl_60 time point comparison but did significantly change in the iz/anl_60-ac/epi_neo time point comparison, and iii) transient: those genes that significantly change during the iz/anl_45-iz/anl_60 time point comparison but then significantly change in the opposite direction during the iz/anl_60-ac/epi_neo time point comparison and iv) consistent: those genes that experience fold change in expression consistently throughout the time series. We also divided each of these whole time series gene sets into positive and negative sets corresponding to the sign of the fold-change. In the case of transient, the directionality corresponds to the fold change in the first, iz/anl_45-iz/anl_60 time point comparison.

To evaluate GOcats’ potential to improve the statistical power of annotation enrichment, we compared early and late time point annotation enrichments derived from GOcats ancestor traversal to the extreme time points annotation enrichment derived from traditional ancestor traversal. Here we define the following sets of annotations for each tissue type evaluated:

$$EarlyUniqueEnrichedTerms_{Gocats}=45\_to\_60_{Gocats}–45\_to\_60_{no\_hp}–Transient_{no\_hp}$$ (8)

The 45_to_60_GOcats_ and 45_to_60_no_hp_ variables are the sets of GO terms identified when comparing the iz/anl_45 time point to the iz/anl_60 time point using GOcats or the traditional ancestor mapping method of ignoring the has_part relation, respectively. Transient_no_hp_ is the set of enriched terms categorized as transient for the whole time series using the traditional ancestor mapping method.

$$EarlySupportedEnrichedTerms=EarlyEnrichedTerms_{GOcats}\cap Consistent_{no\_hp}$$ (9)

Consistent_no_hp_ is the set of enriched terms categorized as consistent for the whole time series using the traditional ancestor mapping method.

$$LateUniqueEnrichedTerms_{Gocats}=60\_to\_neo_{Gocats}-60\_to\_neo_{no\_hp}-Transient_{no\_hp}$$ (10)

The 60_to_neo_GOcats_ and 60_to_neo_no_hp_ variables are the sets of GO terms identified when comparing the iz/anl_60 time point to the ac/api_neo time point using GOcats or the traditional method of ignoring the has_part relation, respectively.

$$LateSupportedEnrichedTerms=LateEnrichedTerms_{GOcats}\cap Consistent_{no\_hp}$$ (11)

### RNASeq analysis of horse cartilage development time points

Tissue samples were collected across six experimental groups (Table 7) and compared for differential gene expression at a transcriptome level using mRNA sequencing. Sample collection methods have been described previously [10,25] and were conducted in accordance with an approved University of Kentucky Institutional Animal Care and Use Committee protocol (# 2014–1215). Total RNA was isolated using a commercial kit (Qiagen RNeasy Micro Kit, cat# 74004) after homogenization on ice as previously described [26]. Following ethanol precipitation and re-solubilization in sterile distilled water, the total RNA was quantified using a fluorometric assay (Qubit, Life Technologies, Q10210, Q32852) and assessed for chemical contaminants using a spectrophotometer (NanoDrop ND 1000) and for structural integrity with a Bioanalyzer 2100 (Agilent Technologies, Eukaryotic Total RNA Nano & Pico Series II). All RNA samples met quality thresholds of 260/280 absorbance ratios of 1.7–2.0, 260/230 absorbance ratios of 1.8–2.1, and an Agilent RNA integrity number (RIN) of ≥ 7.0.

**Table 7:** Comparison of equine fetus tissue samples.

Sample Description		Age	Tissue source
Equine Fetus	Interzone (n = 7)	45–46 days gestation	Carpal and tarsal joints
	Anlage (n = 6)		Metaphysis of distal humerus and femur
Equine Fetus	Interzone (n = 7)	57–66 days gestation	Carpal joints
	Anlage (n = 7)		Metaphysis of distal humerus and femur
Equine Neonate	Articular cartilage (n = 7)	0–9 days postnatal	Femorotibial joint
	Epiphyseal cartilage (n = 7)		Proximal tibia

RNAseq libraries were constructed using the TruSeq HT Stranded RNA Sample Preparation Kit (Illumina San Diego, CA). PolyA+ RNA was selected from 1 μg of total RNA and first-strand synthesis performed using random hexamer primers and SuperScript II reverse transcriptase (Life Technologies). Resulting double-stranded cDNA was then blunt-ended and ligated to indexed adaptors, followed by PCR amplification for 12 cycles with Kapa HiFi polymerase (Kapa Biosystems, Woburn, MA). Libraries were initially quantitated using Quant-it (Life Technologies, Grand Island, NY) and the average size determined on an AATI Fragment Analyzer (Advanced Analytics, Ames, IA). They were then diluted to a final concentration of 5nM and further quantitated by qPCR on a BioRad CFX Connect Real-Time System (Bio-Rad Laboratories, Inc. CA).

Strand-specific sequencing was performed using a paired-end mRNA-seq protocol (http://www.illumina.com/technology/paired_end_sequencing_assay.ilmn) at the Roy J. Carver Biotechnology Center, University of Illinois at Urbana-Champaign. A minimum of 30 million reads were generated for each sample, trimmed (Trimmomatic Version 0.36, http://www.usadellab.org/cms/?page=trimmomatic), and then mapped to the equine reference genome (EquCab2.0, chromosomes 1–31, M, X, and Un, NCBI Annotation Release 102) using MapSplice 3.0 Beta [27]. Default settings were used. Steady state levels of mRNA levels were compared between the six experimental groups at all protein-coding gene loci structurally annotated in the equine genome (EquCab2.0, NCBI Annotation Release 102) by DESeq2 analysis [28]. DESeq2 modeled the read count data using negative binomial distribution and performed the statistical testing for differential gene expression. The analysis returned a p-value determined by Wald statistics and an adjusted p-value (to apply corrections for multiple comparisons testing). The Benjamini-Hochberg multiple-test correction was applied to evaluate the false-discovery rate (FDR). The DESeq2 identified 5572 (ANL_45 to ANL_60), 5464 (ANL_45 to Epi_neo), 7049 (ANL_60 to Epi_neo), 9929 (IZ_45 to IZ_60), 9975 (IZ_45 to AC_neo), and 8329 (IZ_60 to AC_neo) differentially expressed genes, which have an adjusted p-value < 0.01 after multiple testing corrections.

Scripts and snakemake [29] workflows for performing these analyses can be found in Scripts Directory 4 in the FigShare directory available at. https://figshare.com/s/9d55b2e5932992e6a068

## Supporting information

S1 File Comparing adjusted p-values between omitted has_part and GOcats part_of_some edges. (ZIP)
