# Supplementary figures and images for: Advances in gene ontology utilization improve statistical power of annotation enrichment
Source: PLoS One. 2019 Aug 15;14(8):e0220728. doi: 10.1371/journal.pone.0220728 (PMC6695228; doi:10.1371/journal.pone.0220728)

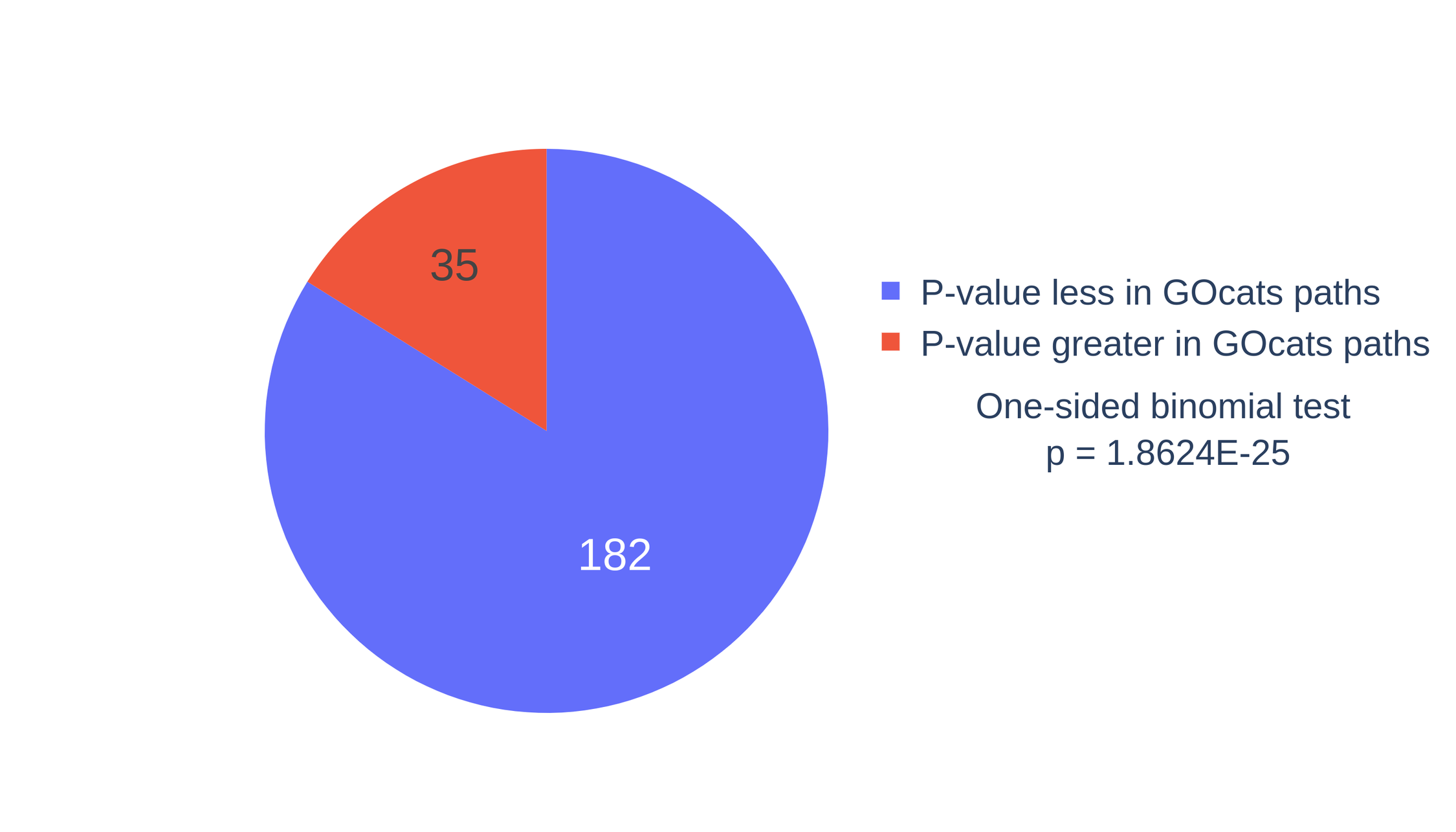

Supplement: S1 File — (ZIP) [file pone.0220728.s001.zip › RelationsFiguresTablesSupplemental/Figure3 - Comparing p-values of significantly-enriched annotations using GOcats paths vs excluding has_part edges.tif]

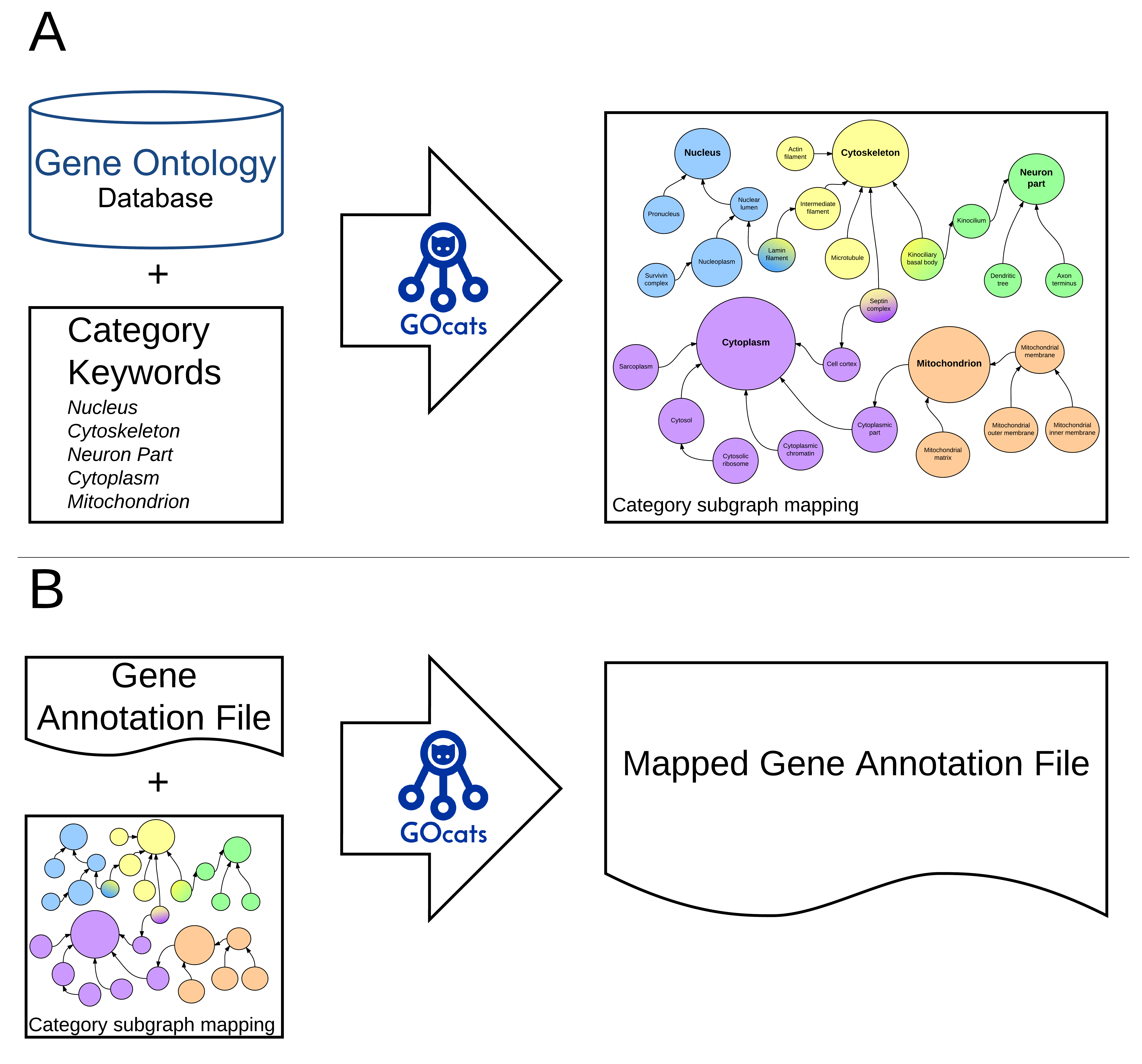

Supplement: S1 File — (ZIP) [file pone.0220728.s001.zip › RelationsFiguresTablesSupplemental/Figure1 - GOcats data flow diagram for subgraph creation and GAF mapping.tif]

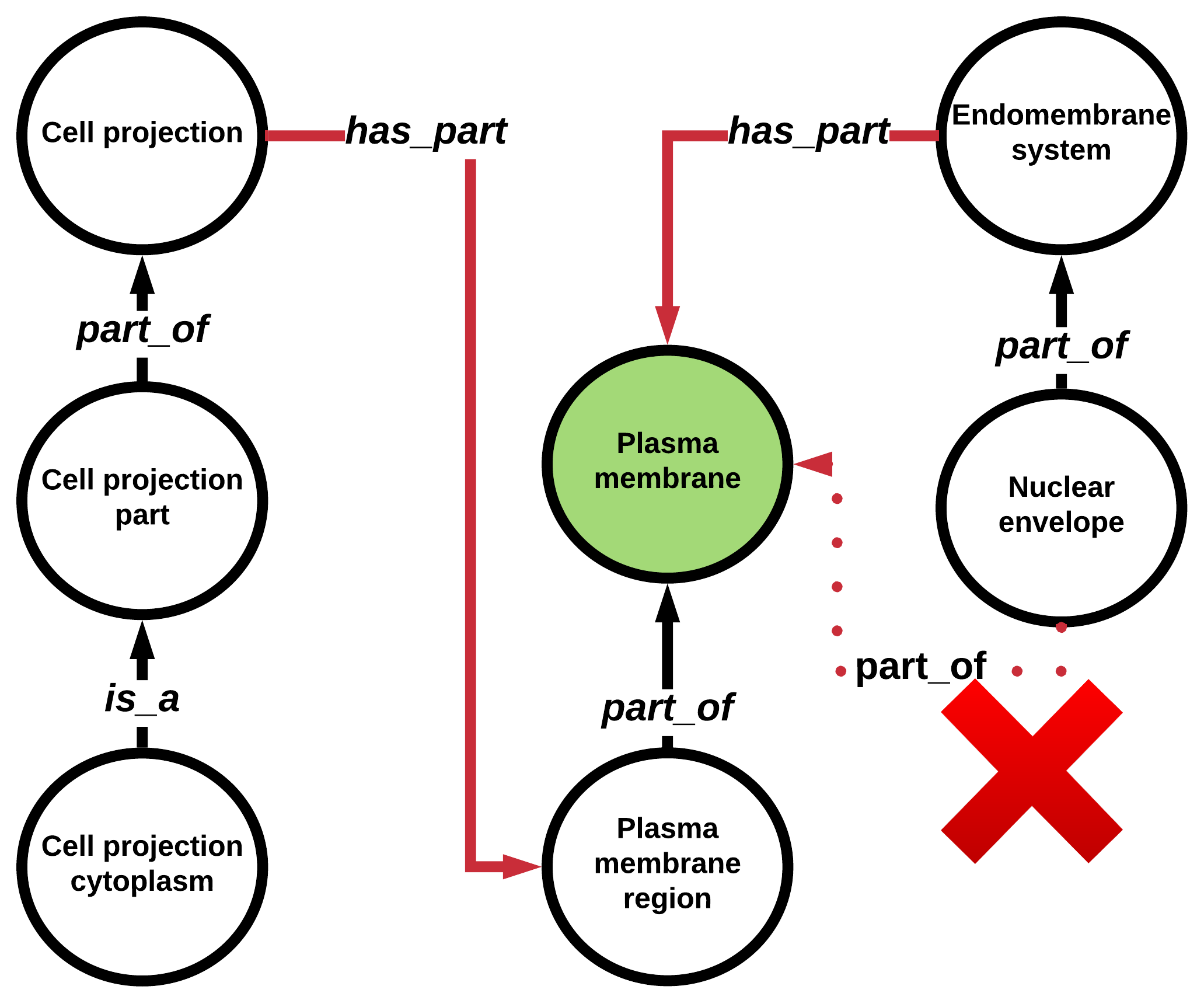

Supplement: S1 File — (ZIP) [file pone.0220728.s001.zip › RelationsFiguresTablesSupplemental/Figure2 - The has_part relation creates paths with of varying semantic scoping which confuses mapping within GO, simplified.tif]
